# Three-dimensional movements of the pectoral fin during yaw turns in the Pacific spiny dogfish, *Squalus suckleyi*

**DOI:** 10.1242/bio.037291

**Published:** 2018-12-24

**Authors:** Sarah L. Hoffmann, Cassandra M. Donatelli, Samantha C. Leigh, Elizabeth L. Brainerd, Marianne E. Porter

**Affiliations:** 1 Florida Atlantic University, Department of Biological Sciences, Boca Raton, FL 33431, USA; 2 Tufts University, Department of Biology, Medford, MA 02155, USA; 3 University of California, Irvine Department of Ecology and Evolutionary Biology, CA 92697, USA; 4 Brown University, Department of Ecology and Evolutionary Biology, Providence, RI 02912, USA; 5 Friday Harbor Labs, University of Washington, Friday Harbor, WA 98250, USA

**Keywords:** Rotation, Maneuvering, Volitional swimming, VROMM

## Abstract

Fish pectoral fins move in complex ways, acting as control surfaces to affect force balance during swimming and maneuvering. Though objectively less dynamic than their actinopterygian relatives, shark pectoral fins undergo complex conformational changes and movements during maneuvering. Asynchronous pectoral fin movement is documented during yaw turning in at least two shark species but the three-dimensional (3D) rotation of the fin about the body axes is unknown. We quantify the 3D actuation of the pectoral fin base relative to the body axes. We hypothesized that Pacific spiny dogfish rotate pectoral fins with three degrees of freedom relative to the body during volitional turning. The pectoral fin on the inside of the turn is consistently protracted, supinated and depressed. Additionally, turning angular velocity increased with increasing fin rotation. Estimated drag on the fin increased and the shark decelerated during turning. Based on these findings, we propose that Pacific spiny dogfish uses drag-based turning during volitional swimming. Post-mortem muscle stimulation revealed depression, protraction and supination of the pectoral fin through stimulation of the ventral and cranial pterygoideus muscles. These data confirm functional hypotheses about pectoral fin musculature and suggest that Pacific spiny dogfish actively rotate pectoral fins to facilitate drag-based turning.

This article has an associated First Person interview with the first author of the paper.

## Abstract (precis)

**Summary:** Pacific spiny dogfish rotate the inside pectoral fin consistently about three axes during yaw turning, which correlates with turning performance. Stimulation experiments confirm hypotheses about muscle function in controlling actuation.

## INTRODUCTION

The morphology and movement of control surfaces (structures that adjust an organism's position in space) in swimming vertebrates have profound effects on stability and maneuverability (Webb and Weihs, 2015; Fish and Lauder, 2017). Paired fins and flippers are particularly important in balancing forces during steady swimming and reorienting force during maneuvering (Harris, 1936; Nursall, 1962; Fish, 1997; Fish and Shannahan, 2000; Fish, 2002; Wilga and Lauder, 2000; Fish et al., 2018). Despite the vast diversity of whole body morphology and swimming styles [i.e. median paired fin (MPF) versus body caudal fin (BCF)], the pectoral fins of fishes are widely acknowledged as dynamic control surfaces generating thrust, lift and drag critical to maneuvering (Webb, 1984; Drucker and Lauder, 2002, 2003; Lauder and Drucker, 2004; Webb and Weihs, 2015; Fish and Lauder, 2017). Synchronous pectoral fin rotation symmetrically alters force generation such that the horizontal swimming trajectory is unaffected. For example, sunfish rotate both pectoral fins to direct forces anteriorly, resulting in reactional braking directed through the center of mass (Drucker and Lauder, 2002). Alternatively, asynchronous pectoral fin rotation generates an imbalance of forces and initiates yawing (horizontal maneuvering). Sunfish and trout rotate the outside fin to generate a laterally oriented force and turn the body horizontally, while the inside fin directs thrust posteriorly moving the fish forward (Drucker and Lauder, 2002; Lauder and Drucker, 2004). Asynchronous pectoral fin movement is also observed in shark yaw turning (Kajiura et al., 2003; Domenici et al., 2004), but the 3D kinematics and their effect on turning have not been quantified.

The volitional swimming behavior of sharks has been documented in a few species but is limited by use of 2D video (Lowe, 1996; Kajiura et al., 2003; Domenici et al., 2004; Porter et al., 2009; Porter et al., 2011; Hoffmann et al., 2017). Studies examining yaw maneuvering in sharks used dorsal video and focused on whole body kinematics, but asynchronous pectoral fin movement has also been noted during turning (Kajiura et al., 2003; Domenici et al., 2004). In a dorsal view of the bonnethead shark, the visible surface area of the pectoral fin area inside the body curvature is significantly smaller than the outside fin, suggesting the pectoral fins may play different roles during turning (Kajiura et al., 2003). Similarly, the spiny dogfish differentially moves the pectoral fins to create tight turning radii during escape maneuvers (Domenici et al., 2004). In these instances, fin movement is hypothesized to increase drag, thereby creating a turning moment (Kajiura et al., 2003). Despite observations that they are dynamic control surfaces rotating on at least two axes, the role of pectoral fin movement in yaw maneuvering remains unclear (Pridmore, 1994; Wilga and Lauder, 2000, 2001; Kajiura et al., 2003; Domenici et al., 2004; Oliver et al., 2013).

The pectoral fins of fishes become increasingly mobile and flexible through evolutionary time, yet the fins of basal clades are also described to have some range of motion in relation to the body (Wilga and Lauder, 1999; Lauder, 2015). The kinematics and morphology of shark pectoral fins are described in a few species, and are generally stiffer than those of ray finned fishes and lack jointed fin rays. Though shark pectoral fins undergo substantial conformational changes during swimming, they are not collapsible to the same degree as most actinopterygian fins (Fish and Shannahan, 2000; Wilga and Lauder, 2000, 2001; Lauder, 2015). Despite this major difference in flexibility and structure, shark pectoral fins are mobile at the insertion and have associated musculature that is well situated to actuate 3D rotation of the fin in relation to the body (Marinelli and Strenger, 1959; Fish and Shannahan, 2000; Wilga and Lauder, 2000, 2001). Squalids have three pectoral fin muscles associated with the pectoral fin: the cranial pterygoideus (CP), dorsal pterygoideus (DP) and ventral pterygoideus (VP), which are hypothesized to protract, elevate and depress the fin, respectively (Marinelli and Strenger, 1959). Previous data on leopard sharks, *Triakis semifaciata*, demonstrate that during vertical maneuvering, the dorsal and ventral fin muscles are active during rising and sinking as the fin elevates and depresses, respectively (Maia et al., 2012). We hypothesize that active fin rotation about the pectoral girdle plays a major role in reorienting the fin, and thus force generation, during maneuvering.

One factor that confounds the role of pectoral fin rotation in shark maneuvering is the use of differing anatomical and rotational terminology (Pridmore, 1994; Liem and Summers, 1999; Goto et al., 1999; Fish and Shannahan, 2000; Wilga and Lauder, 2000, 2001; Kajiura et al., 2003; Oliver et al., 2013). During vertical maneuvering, the pectoral fins are described as ‘ventrally rotated’ or changing angle of attack to flow, which may refer to both depression or pronation/supination of the fin (Fish and Shannahan, 2000; Wilga and Lauder, 2000, 2001). Additionally, depression/elevation are often used interchangeably with abduction/adduction to describe pectoral fin rotation about the rostro-caudal axis (Table 1; Marinelli and Strenger, 1959; Liem and Summers, 1999; Wilga and Lauder, 2001; Oliver et al., 2013). For example, Oliver et al. (2013) noted that the pelagic thresher, *Alopias pelagicus*, adducts both pectoral fins to initiate a breaking moment during tail slaps. Put in context, the fins are likely depressed, though fin depression has also been referred to as abduction (Table 1; Wilga and Lauder, 2001; Oliver et al., 2013). Thresher fin movement is further described as ‘laterally rotated’, potentially referring to either rotation of the fin about the rostro-caudal axis (elevation) or the dorso-ventral axis (protraction/retraction) (Oliver et al., 2013). Qualitative observations of pectoral fin movement describe fins as being ‘tucked’ under the bonnethead shark during turning, and ‘swinging’ during walking in the epaulette shark (*Hemiscyllium ocellatum*), but the 3D movement of the fin remains unclear (Pridmore, 1994; Goto et al., 1999; Kajiura et al., 2003). Resolving the terminology used to describe pectoral fin movement specific to sharks will greatly increase our understanding of their functional roles and associated musculature.

**Table 1.**
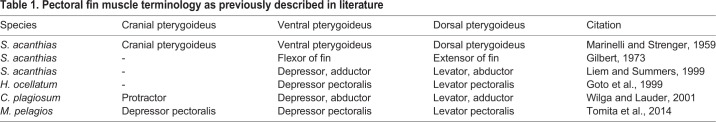
**Pectoral fin muscle terminology as previously described in literature**

The goal of the present study is to describe 3D movement of Pacific spiny dogfish pectoral fins during routine yaw turning and under targeted muscle stimulation. We aimed to (1) quantify the 3D rotations of pectoral fins in relation to the body, (2) investigate the effects of pectoral fin movement on whole body maneuvering kinematics, and (3) describe pectoral fin rotation in response to targeted stimulation of pectoral girdle musculature. In free-swimming sharks, we targeted yaw turns since previous studies documented pectoral fin movement during horizontal maneuvering and proposed that pectoral fin depression generates turning momentum (Kajiura et al., 2003; Domenici et al., 2004). Similarly, we hypothesized that the fin inside the body curvature would be depressed to generate torque during turning. Swimming trials were followed with targeted post-mortem muscle stimulation to determine the role of pectoral girdle musculature in fin actuation. We hypothesized that post-mortem muscle stimulation of the DP, VP and CP would result in elevation, depression and protraction of the fin, respectively.

## RESULTS

### Pectoral fin kinematics during routine turns

**Fig. 1.**
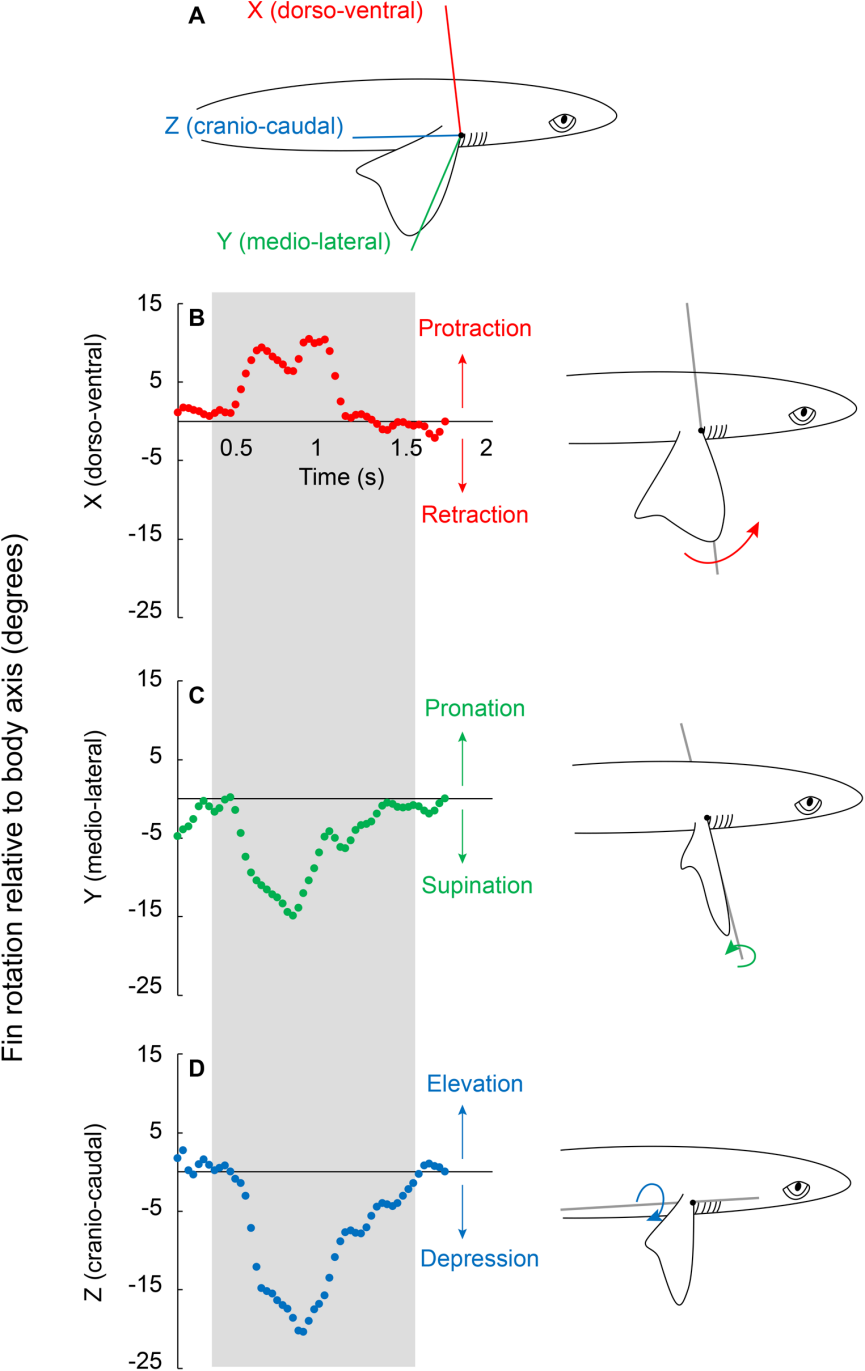
**Pectoral fin rotation relative to the body axes.** (A) The 3D shapes represent the body and inside fin. A joint coordinate system (FB-JCS) was placed at the proximal fin base to measure relative fin rotation about the dorso-ventral (X; red), medio-lateral (Y; green), and cranio-caudal (Z; blue) axes. (B–D) Sample rotation trace of one turn highlighted in the gray box. The pectoral fin was (B) protracted, (C) supinated and (D) depressed during the turn. This pectoral fin rotation pattern was observed in all nine trials. Protraction rotates the fin cranially, supination causes the trailing edge of the fin to translate ventrally, increasing the angle of attack, and depression makes the negative dihedral angle of the fin more negative.

**Fig. 2.**
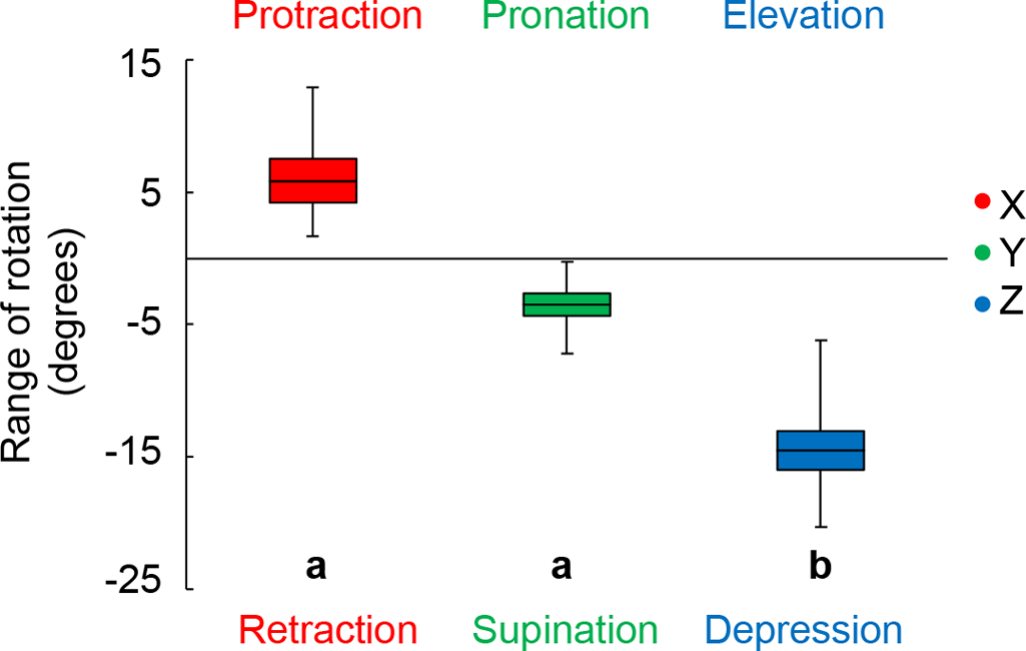
**We found significantly greater depression of pectoral fins during turning.** For all nine trials, the inside pectoral fin was protracted, supinated and depressed. The absolute values of rotation were used in an ANOVA to compare the range of rotation in each axis and lower-case letters denote significant differences. The box represents the mean (middle line)±s.e. of the mean. Whiskers represent the upper and lower extremes. F2,24=20.6537, P<0.0001.

**Fig. 3.**
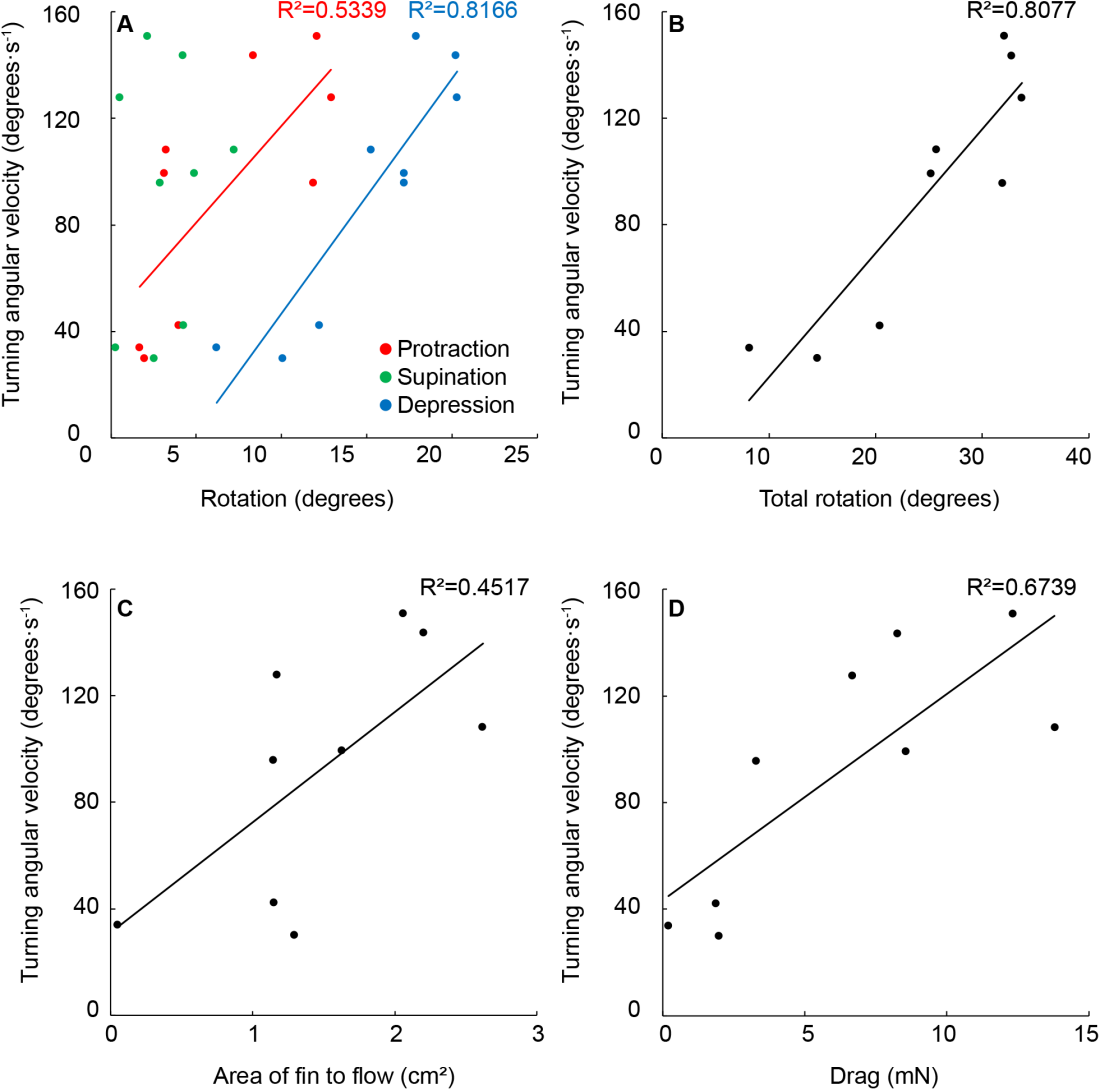
**Relation among pectoral fin movement and turning kinematics.** (A) Both depression and protraction were positively related to turning angular velocity while supination was not (*P*=0.253, *P*=0.0008). (B) When considering the sum of rotations about all three axes, there was a significant positive relation with turning angular velocity (*P*=0.0010). (C) The area of fin to flow was significantly positively related to turning angular velocity (*P*=0.0474). (D) Drag was also significantly positively correlated with turning angular velocity (*P*=0.0098).

For all trials (*n*=9), the inside fin was protracted, supinated and depressed while the shark executed yaw turns (Movie 1) (Fig. 1). We report all variables from the frame of maximum total rotation. Maximum pectoral fin rotation about the X axis ranged from 2° to 13° (Figs 1B and 2; 6.6±1.57°). Y axis rotation ranged from −0.3° to −7° (Figs 2C and 3; −4.5±0.83°); and the greatest rotation was about the Z axis, which ranged from −6° to −20° (Figs 1D and 3; −15.6±1.47°; *F*_2,24_=20.6537, *P*<0.0001). Total fin rotation (the sum of the absolute value of rotation in all three axes) ranged from 8° to 34° (27.6±1.98°).

Inside fin depression and protraction were both positively related to shark turning angular velocity (Fig. 3A; R^2^=0.5339, *P*=0.0253; R^2^=0.81662, *P*=0.0008; respectively). Total fin rotation (*β*), calculated as the sum of rotation in all three axes, was positively related to shark turning angular velocity (Fig. 3B; R^2^=0.8077, *P*=0.0010). To estimate drag, we used fin rotation to determine the projected area of the fin to flow. The mean area of fin to flow, derived using fin rotation, was 1.5±0.25 cm^2^ (Fig. 3C). We also calculated instantaneous velocity throughout the trial to estimate force (Eqn 3). In every trial, we found that velocity (Δ*V*) decreased during the turn (−10.0±1.30 cm s^−1^). We estimated instantaneous drag throughout each trial (Fig. 3D; 6.32±0.16 mN). Area of the fin to flow (*A*) and drag (*F_d_*; Eqn 3) were also significantly positively related to shark turning angular velocity (Fig. 3C,D; R^2^=0.4517, *P*=0.0474; R^2^=0.6379, *P*=0.0098).

### Pectoral fin muscle stimulation

**Fig. 4.**
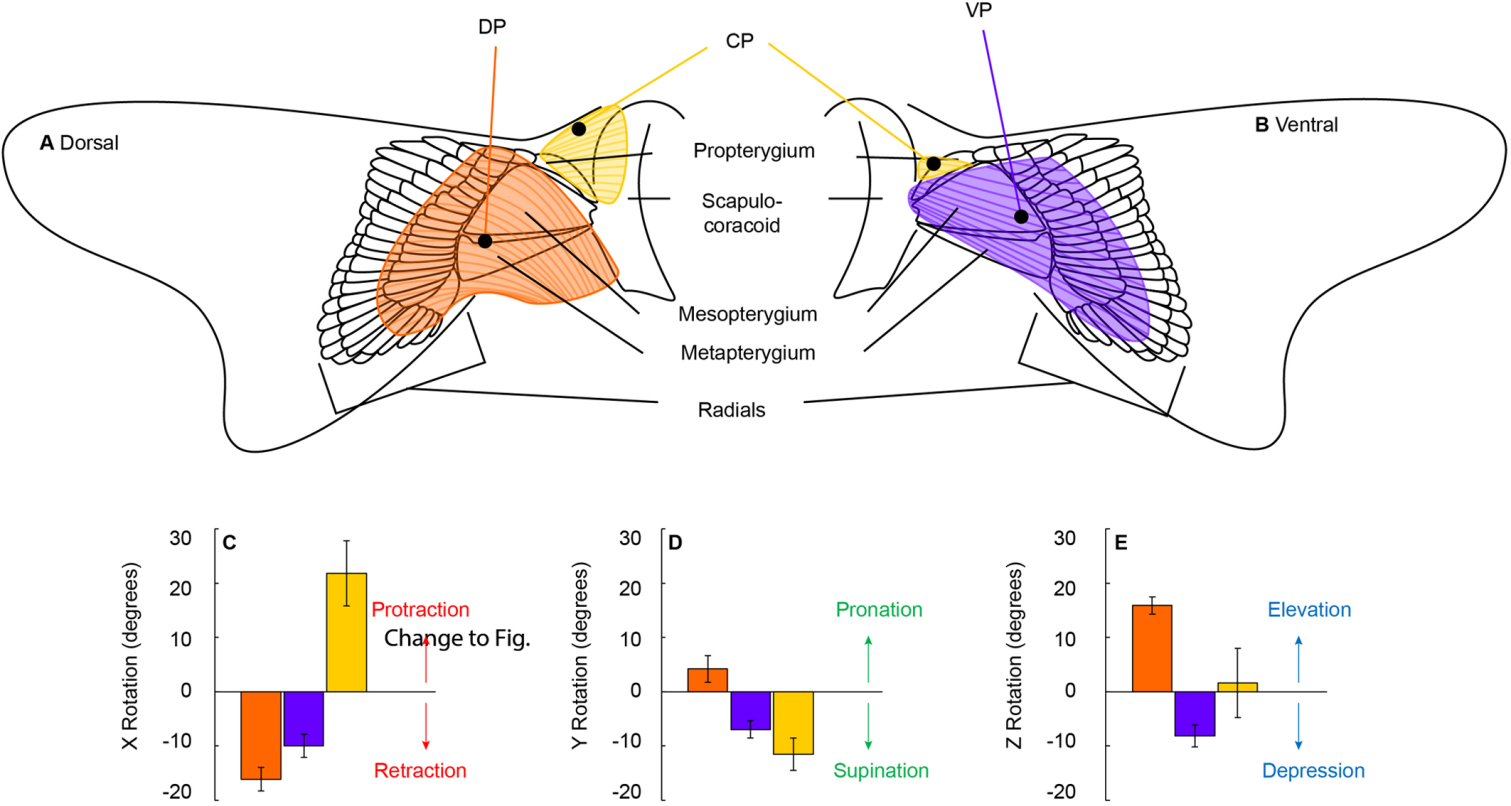
**Skeletal and muscular morphology of the pectoral fin of the Pacific spiny dogfish.** Three basal cartilages (propterygium, mesopterygium and metapterygium) articulate with the scapulo-coracoid. Radial elements fan out distally and support the fin web. (A) On the dorsal side of the fin, the DP originates on the scapulo-coracoid and inserts distally on the intermediate radials. The CP originates on the dorsal margin of the scapula-coracoid and inserts on both the dorsal and (B) ventral portions of the propterygium. The VP originates on the ventral margin of the scapula-coracoid and inserts ventrally on the intermediate radials. Black dots mark the target of lead placement for each muscle. (C–E) Post mortem muscle stimulation resulted in fin rotation about the three body axes described in Fig. 2. Only one lead was placed per muscle, but placement in the CP was along the anterior margin of the leading edge and can be seen in the dorsal and ventral view. (C) The CP was the only muscle to result in protraction when stimulated. (D) Stimulation of the VP and CP resulted in supination whereas stimulation of the DP pronated the fin. (E) The DP elevated the fin when stimulated and the VP depressed the fin, supporting the hypothesis that they are antagonistic muscles.

Targeted stimulation of the DP muscle resulted in 16.1±2.16° of retraction, 4.1±2.50° of pronation, and 15.9±1.58° of elevation (Fig. 4). Similarly, stimulation of the VP resulted in 10.0±2.12° of retraction; however, the fin was also supinated (7.0±1.61°) and depressed (8.2±2.03°; Fig. 4). The only muscle stimulation to result in protraction was the CP (21.9±5.98°; Fig. 4). Stimulation of the CP also resulted in 11.5±3.00° of supination, and no definitive trend for depression or elevation. The CP originates on the scapula-coracoid and inserts on both the dorsal and ventral portion of the propterygium. Dissections confirmed that leads were placed in the CP for all three individuals, but placement within the CP was variable. In one individual, lead placement was slightly dorsal to the propterygium, and in the two other sharks, leads were slightly ventral to the propterygium. Variable placement in the CP resulted in 14.43° of elevation in the dorsally placed lead, while stimulation in the other two individuals resulted in depression (8.1±3.27° s.e.m.).

## DISCUSSION

We found that Pacific spiny dogfish rotate the inside pectoral fin substantially about all three axes in a consistent manner while executing yaw turns (Fig. 2). These rotations turned the fin to increase the area to the flow, likely increasing drag. Targeted stimulation of pectoral fin muscles further suggests that pectoral fin rotation is under muscular control (Fig. 4). These three results together suggest that sharks actively protract, supinate and depress the inside pectoral fin to facilitate drag based yaw turning (Figs 2 and 3).

### Pectoral fin rotation during volitional turning

Shark pectoral fins are dynamic control surfaces that adjust position and conformation during vertical maneuvering (Daniel, 1922; Harris, 1936; Alexander, 1965; Fish and Shannahan, 2000; Wilga and Lauder, 2000; Maia et al., 2012). Our data further document pectoral fin rotation, now in the context of yaw maneuvering (Figs 2 and 3). We found that the inside fin is depressed up to 20° in all trials and both pectoral fin depression and protraction (up to 12°) were significantly related to the turning angular velocity of the shark (Figs 2 and 3A). Total pectoral fin rotation was also significantly related to turning angular velocity, suggesting that fin rotation may contribute to the turning speed of the shark (Fig. 3B). Total pectoral fin rotation doubled between the slowest and fastest turns recorded, with a fivefold increase in the turning angular velocity (Fig. 3B). This study describes volitional turning and the variability in these data likely reflects the range of behaviors seen in a natural environment. The range of data presented here demonstrates that pectoral fins rotate about all three axes and there was a strong relationship between total fin rotation and turning angular velocity (Fig. 3).

From the maximally-rotated 3D pose of the fin in each trial we calculated the area of the fin to flow, which was positively related to turning angular velocity (Fig. 3C). We found a significant relation between the turning angular velocity, total fin rotation and estimated drag, consistent with the hypotheses that fin rotation generates drag to aid in yaw maneuvering (Fig. 3D; Kajiura et al., 2003; Domenici et al., 2004). We also noted a decrease in velocity during turning in each trial which, in combination with high drag estimates, suggests that pectoral fin rotation contributed to drag based turning (Kato et al., 2004; Fish and Nicastro, 2003; Fish and Lauder, 2017). The pectoral fin drag we estimate in this study is similar to that previously measured for bamboo sharks during vertical maneuvering and slightly less than drag measured on robotic flapping fins (Wilga and Lauder, 2001; Tangorra et al., 2010). We propose that increasing the area of the fin to flow generates drag, causing the fin to act as a pivot about which the body rotates (Fig. 3D). In this study, we describe pectoral fin rotation in relation to turning performance; but axial bending, caudal fin movement, and dorsal fin stiffening may all play substantial role in yaw maneuvering (Kajiura et al., 2003; Domenici et al., 2004; Porter et al., 2009; Maia and Wilga, 2013). Further studies considering whole body kinematics would greatly increase our understanding of the forces acting on the body during yaw maneuvering.

Pectoral fin movement and function has been previously described in many ways but lacks standardized terminology. For example, epaulette sharks are described as ‘swinging’ their pectoral fins during walking, bonnethead sharks ‘tuck’ their fins during turning, and pelagic thresher sharks are noted to ‘laterally rotate’ their fins during braking (Pridmore, 1994; Kajiura et al., 2003; Oliver et al., 2013). Additionally, the use of ‘adduction’ and ‘abduction’ is interchanged throughout the literature, and it is less applicable to the position and movement of shark pectoral fins compared to ray finned fishes (Table 1; Marinelli and Strenger, 1959; Wilga and Lauder, 2001; Oliver et al., 2013). Our findings demonstrate that shark pectoral fins rotate about three axes, and we propose the use of protraction/retraction, pronation/supination and depression/elevation to standardize the description of movements, similar to terminology used to describe wing rotation in bird flight (Fig. 1; Dial et al., 1988; Tobalske et al., 2003; Tobalske, 2010). These motions are all alluded to in previous studies with varying terminology. For example, epaulette sharks use sequential fin protraction/retraction in a walking gate (Pridmore, 1994; Goto et al., 1999). Shark fin depression and elevation are described previously as the change in their negative dihedral angle of to the body and may also be referred to as adduction/abduction (Table 1; Alexander, 1965; Ferry and Lauder 1996; Fish and Shannahan, 2000; Wilga and Lauder, 2000, 2001). Differences in fin elevation/depression and pronation/supination is the least clear since many previous studies describe changes in fin angle of attack, which may be a result of either of these motions or some combination. Here, we propose the use of pronation/supination to describe the long axis rotation of the fin where pronation results in the dorsal movement of the trailing edge relative to the leading edge and supination vice-versa. In this instance, ninety degrees of rotation in either direction would result in the trailing edge being directly in line with the leading edge.

In this study, we examined one species assuming that the base of the pectoral fin acted roughly as a rigid body. Shark pectoral fin morphology is variable and fins undergo behavior mediated conformational changes during swimming and maneuvering that would substantially affect force generation (Moss, 1972; Wilga and Lauder, 2000, 2001; Maia et al., 2012). We suggest caution should be taken when generalizing functional roles among all species and fin morphologies. Further endeavors to validate this role would benefit from integrating volitional swimming with particle image velocimetry, and computational fluid dynamics in a comparative context.

### Pectoral fin musculature

We showed that pectoral fins rotate during yaw maneuvering, and we used post-mortem muscle stimulation experiments to show that fins are under muscular control. We found that contraction of the VP resulted in 8° of pectoral fin depression and contraction of the DP resulted in 16° of elevation (Fig. 4E). These experimental results support functional hypotheses derived from anatomical descriptions of pectoral fin muscles (Marinelli and Strenger, 1959; Liem and Summers, 1999; Goto et al., 1999; Wilga and Lauder, 2001). Volitional swimming trials showed that the inside fin is depressed up to 27°, and post-mortem stimulation resulted in 8° of depression in the VP and also in the CP, when the lead was placed in the ventral propterygium. We hypothesize that simultaneous activation of the VP and CP resulted in greater pectoral fin depression during volitional swimming.

In addition to fin depression, we found that stimulation of the CP was solely responsible for 22° of pectoral fin protraction (Fig. 4C). The CP fans out over the anterior portion of the propterygium, located on both the dorsal and ventral margins of the fin with muscle fibers oriented orthogonally to the body axis (Marinelli and Strenger, 1959; Liem and Summers, 1999; Goto et al., 1999; Wilga and Lauder, 2001). In volitional turning, the maximum amount of pectoral fin protraction we observed was 18°, and we hypothesize that the CP is the only muscle responsible for controlling pectoral fin protraction. Alternatively, stimulation of the DP and VP resulted in 16° and 10° of pectoral fin retraction; respectively (Fig. 4C). The muscle fibers of the DP and VP are at an oblique angle to the body fanning out posterolaterally, resulting in pectoral fin retraction, along with rotation in other planes (Marinelli and Strenger, 1959; Liem and Summers, 1999; Wilga and Lauder, 2001; Fig. 4). Stimulation of all three pectoral fin muscles resulted in long axis rotation: stimulation of the CP and VP resulted in 7° and 9° supination; respectively; and stimulation of DP resulted in 4° of pronation (Fig. 4D). Previous studies note changes in pectoral fin angle of attack, resulting from depression/elevation, pronation/supination, or some combination thereof, but the resulting fin rotation in these experiments is unclear (Fish and Shannahan, 2000; Wilga and Lauder, 2000, 2001).

We targeted intrinsic pectoral fin muscles contained within the fin, but there is also extrinsic axial musculature associated with the fin that may contribute to rotation (Marinelli and Strenger, 1959; Liem and Summers, 1999; Wilga and Lauder, 2001). The cucullaris originates on the scapular process of the pectoral girdle and runs longitudinally to insert on anterior epaxial muscles, and it is hypothesized to protract the scapula (Marinelli and Strenger, 1959; Liem and Summers, 1999; Wilga and Lauder, 2001). Additionally, a portion of the hypaxialis inserts on the posterior margin of the scapular process and may play a role in retracting the pectoral girdle (Marinelli and Strenger, 1959; Liem and Summers, 1999; Wilga and Lauder, 2001). Recent work demonstrated that the pectoral girdle of white-spotted bamboo sharks is mobile during suction feeding, and it is hypothesized that this movement may also occur during locomotion (Camp et al., 2017). Future studies on fin actuation should consider the role of the pectoral girdle and associated musculature.

### 3D volitional kinematics

Swimming studies are often conducted in flumes to minimize non-steady locomotion and calibration error, but the unidirectional flow and working volume constraints limit the study of maneuvering and larger-bodied animals. The 3D kinematics of maneuvering behaviors are especially understudied, likely due to problems with calibrating large volumes. Volitional swimming and maneuvering has been studied in a number of large aquatic vertebrates, but is limited by the use of two-dimensional (2D) video, which may oversimplify or disregard movements not visible in the filming plane (Lowe, 1996; Blake et al., 1995; Fish, 1997; Fish and Shannahan, 2000; Rohr and Fish, 2002; Kajiura et al., 2003; Domenici et al., 2004; Porter et al., 2009; Porter et al., 2011; Seamone et al., 2014; Hoffmann et al., 2017). Recent studies have successfully demonstrated the use of multi-camera systems calibrated for 3D analysis in large volume environments (Ros et al., 2011; Sellers and Hirasaki, 2014; Jackson et al., 2016; Fish et al., 2018). Many advances in these techniques were developed using consumer-grade cameras and free open-source software, making the ability to study 3D kinematics increasingly accessible (Hedrick, 2008; Brainerd et al., 2010; Jackson et al., 2016; Knörlein et al., 2016). With our growing understanding of the importance of control surfaces in swimming, data on the 3D kinematics of fins and flippers will greatly benefit fields such as movement ecology and ecomorphology.

**Fig. 5.**
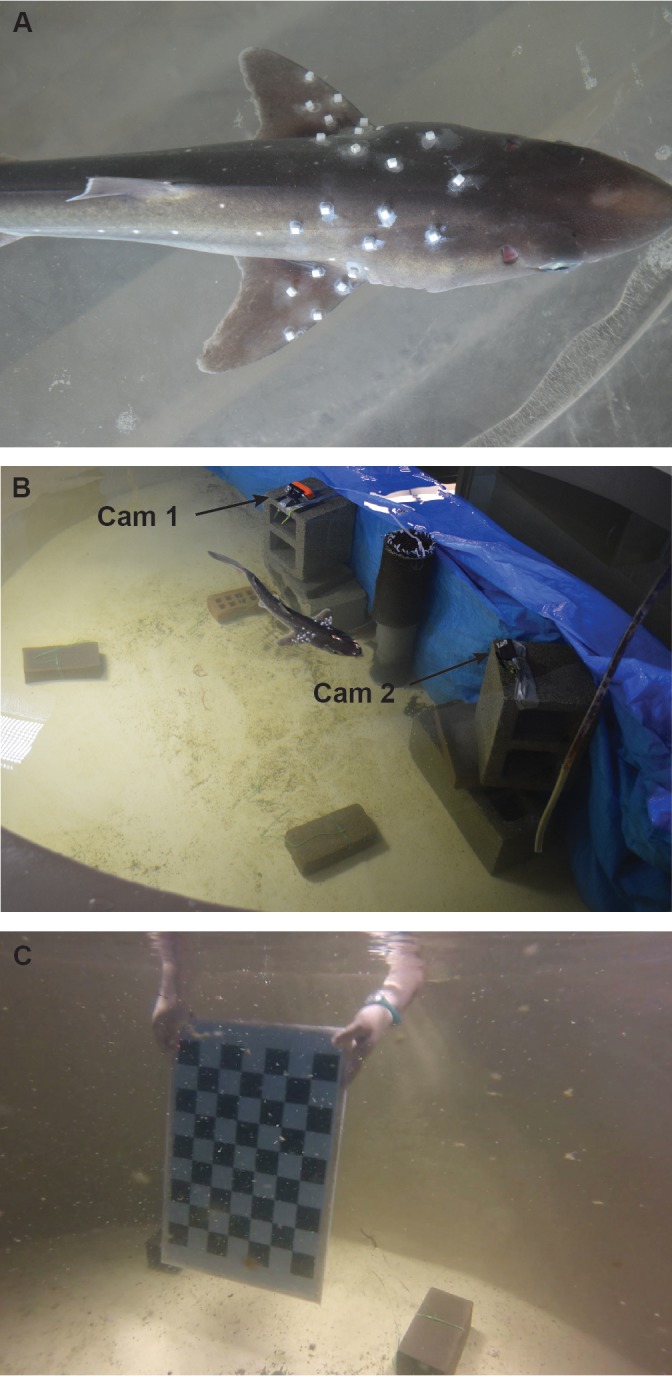
**Video reconstruction of moving morphology (VROMM) experimental design.** (A) Pacific spiny dogfish were outfitted with white bead markers along the anterior body and leading edge of the pectoral fin. (B) Two fully submerged Go-Pro cameras were angled approximately 45° to one another and focused on the same 1 m^3^ volume outlined with bricks. (C) Cameras were time synchronized with a flashing light and calibrated for 3D analyses with a 7×9 checkerboard calibration object.

In this study, we adapted Video Reconstruction of Moving Morphology (VROMM) in two major ways: for use with low-cost, underwater light cameras and fully submerged in a large volume environment (Fig. 5; Brainerd et al., 2010; Knörlein et al., 2016; Jimenez et al., 2018). Marker tracking error, which was on average 0.684 mm [less than 0.02% of the animal's total length (TL)], demonstrates that this method is capturing these larger scale underwater movements with good precision for the size of the arena and size of the animals (Table 2). Further development of this technique has the potential to eliminate size and behavioral constraints that have previously limited the study of volitional movements and prevented 3D motion analysis.

**Table 2.**
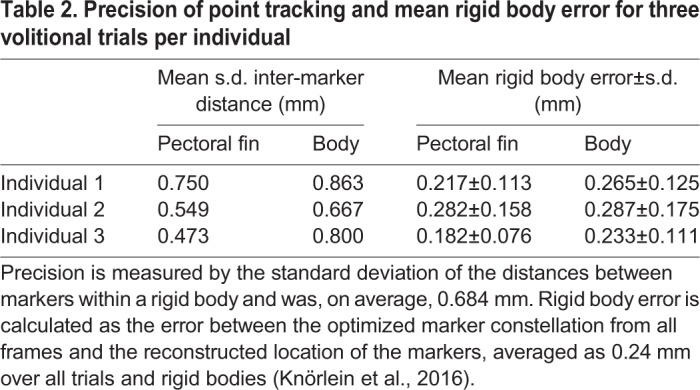
**Precision of point tracking and mean rigid body error for three volitional trials per individual**

### Concluding remarks

The control surfaces of sharks have been largely thought to play a major role in balancing forces on the body during swimming to maintain vertical position in the water column. Here, we demonstrate that pectoral fins move substantially in ways that facilitate maneuvering. Though to a lesser degree, this is similar to the highly mobile pectoral fins of ray finned fishes that are well documented to play a major role in maneuvering. The pectoral fins of sharks and basal actinopterygians also undergo conformational changes during pitch adjustment, further exemplifying the complex movement that fins are capable of in these groups. As with ray finned fishes, physiological and ecological demands have led to a vast diversity of body and fin shape among sharks. Future endeavors should examine the relationship between fin shape and function to better understand the evolution of sharks.

## MATERIALS AND METHODS

Pacific spiny dogfish, *Squalus suckleyi* (*n*=3, 51.2 cm–56.3 cm fork length), were collected via otter trawl in Friday Harbor, WA, USA. All husbandry and procedures were approved under University of Washington IACUC protocol 4239-03. Individuals acclimated in a 2 m diameter tank for a minimum 7 days prior to swimming trials.

### Volitional swimming trials

Individuals were anesthetized via submersion in a 190 l aquarium with a 0.133 g l^−1^ MS-222 solution buffered with NaOH via recirculating aquarium powerhead. White rectangular plastic beads (4×3.5×3.5 mm) were affixed with cyanoacrylate along both the anterior trunk and pectoral fins of the shark (Fig. 5A). Beads were placed so that each region had a minimum of five markers evenly spaced along the semi-rigid parts of the fin and body. After bead placement, which lasted less than 5 min, the individual recovered in a 1.5 m diameter holding tank until normal ventilation resumed. The individual was then transferred to a 2 m diameter, semicircular filming arena with water depth approximately 1.5 m.

Two GoPro Hero3+ light cameras were mounted on cement blocks and positioned along the diameter of the tank, approximately 45° to one another, with the lenses fully submerged (Fig. 5B). Cameras were synchronized with a flashing light, filmed at 30 frames per s (fps) with 1080 p resolution, and were set to narrow field of view. A 1 m^3^ volume was outlined using corks affixed to bricks in the view of both cameras and was calibrated for 3D analyses with a 31.5 cm×40.5 cm checkerboard (Fig. 5C). A minimum of 12 images of the checkerboard calibration object from different regions throughout the volume of interest were tracked for both camera views in XMALab v.1.3.9 to generate a 3D calibration (Knörlein et al., 2016). Any remaining video distortion was corrected in ProDrenalin v.1.0 camera optimization software designed to remove barrel distortion specific to the GoPro Hero 3+ cameras (proDAD, Inc.). After distortion correction, additional images of the calibration object from different regions of the volume of interest were tracked in XMALab and the distance between known points on the checkerboard were calculated and compared to known distances to ensure that any barrel distortion was removed (Knörlein et al., 2016). The difference between the known distance on the checkerboard and calculated distance from tracked points in XMALab did not exceed 0.5 mm.

After resuming normal swimming behavior, individuals were enticed to execute yaw turns in the calibrated volume by placing an object in the path of the shark (Domenici et al., 2004). Total filming time lasted no longer than 3 h, and videos were later clipped into trials in which the shark turned in the calibrated volume. Three trials were selected for each of the three individuals, for a total of *n*=9 trials. Trials were selected in which the fin interior to body curvature (hereafter referred to as the inside fin) and trunk markers are clearly visible, limited change in altitude was observed, and the shark executed a clear yaw turn.

### Muscle stimulation trials

Following volitional swimming trials, individuals were euthanized via submersion in a 2 g l^−1^ MS-222 solution buffered with NaOH. Bipolar electrodes made from 57 μm diameter insulated alloy wire were implanted sub-dermally in post mortem individuals via 21 gauge needles (Flammang, 2010). We targeted pectoral fin muscles and one lead was placed in each of the following: CP (protractor), DP (levator) and VP (depressor) (Fig. 4). Individuals were fully submerged and suspended using a mesh sling in a 190 liter aquarium filled with seawater. Two Panasonic Lumix cameras were positioned 45° to one another and approximately 60 cm away from the tank wall. Cameras were synchronized with a flashing light and the tank volume was calibrated with a 3D calibration object made from Lego bricks (Knörlein et al., 2016). We conducted stimulation trials on one fin for each individual, with one trial per each of the three muscles. Trials were filmed at 120 fps and 1080 p resolution. Electrodes were stimulated individually using a BK Precision 4052 signal generator. A continuous 30 Hz, 10 V square wave pulse was generated until the fin came to rest in the rotated position at which point the pulse was discontinued and the fin returned to resting position (Flammang, 2010). Leads were placed as close to the middle of the muscle as possible and we used a relatively high stimulation to capture maximal muscle recruitment. All pulses lasted less than 2 s, and we observed that the fin was at rest before further stimulation trials on subsequent muscles were conducted.

### 3D marker tracking

Bead markers were tracked in 3D using XMALab v.1.3.9. Rigid bodies were created from a minimum of five beads on the inside fin and trunk. The precision of marker tracking in XMALab is calculated as the standard deviation of the distance between markers in a rigid body, and precision is ±0.1 mm or better in marker based X-ray Reconstruction of Moving Morphology (XROMM) studies (Brainerd et al., 2010; Knörlein et al., 2016). Rigid bodies are typically generated from one bone; however, sharks lack rigid skeletal elements for the formation of true rigid bodies and pectoral fins undergo conformational changes during swimming (Wilga and Lauder, 2000). Even so, the cartilaginous elements of the fin base are well calcified, and we assume that the fin base acts roughly as a rigid body. Further, markers were attached to the skin and there may be some artifact of soft tissue movement that increases marker tracking error (Leardini et al., 2005). For the purposes of this study, we positioned beads at the proximal pectoral fin base towards the leading edge, where the radials support the fin and are tightly associated with a network of collagenous fibers (Fig. 1; Marinelli and Strenger, 1959). Beads were placed in a constellation pattern with a minimum of four beads per rigid body to best describe the mobility of the pectoral fin base relative to the body axes (Knörlein et al., 2016). We treated the fin and trunk region as rigid bodies as an estimation of whole fin movement, since shark pectoral fins change conformation during swimming (Maia et al., 2012; Wilga and Lauder, 2000, 2001). The average standard deviation of inter-marker distance in the fin and trunk rigid bodies in this study was 0.68 mm (Table 2), setting an upper limit of less than 0.02% of body length for the amount of non-rigidity displayed by the fin and trunk in the region of our bead sets. The inter-marker distance errors measured here are larger than the 0.1 mm error that is standard in marker based XROMM (Brainerd et al., 2010) and could be due to the combined effects of non-rigidity and non-homogeneity of material in the fin and body, external placement of markers to skin, use of larger markers and an increase in the volume of interest and associated increase in calibration error.

### VROMM animation

In XMALab, rigid body movement was calculated from the XYZ coordinates of the constellations of markers on the fin and body and the rigid body transformations were filtered using a low-pass Butterworth filter with a 10 Hz cut-off. Rigid body transformations were applied to polygonal mesh shapes representing the fin and trunk in Autodesk Maya 2016 (San Rafael, USA) (Fig. 3A). To measure the motion of the pectoral fin relative to the body, we used the XROMM Maya Tools (xromm.org) to assign a joint coordinate system (JCS) to the articulation point between the fin base and body (FB-JCS; Movie 1; Camp and Brainerd, 2014, 2015). FB-JCSs were placed to minimize translation and measure the three degrees of rotational freedom of the inside fin in relation to the body. FB-JCSs were oriented so that the Z-axis was directed cranio-caudally, the Y-axis medio-laterally and the X-axis dorso-ventrally (Fig. 2A). The FB-JCS rotations were calculated as Euler angles with the rotation order Z,Y,X. JCSs measure rotation according to the right-hand rule so that positive rotation about the axes represents the following anatomical movements of the inside fin: Z-axis elevation, Y-axis pronation and X-axis protraction (Fig. 2A).

### Data analysis

Pectoral fin movement was described as the range of Euler angle rotation (α; deg) about the three FB-JCS axes when the individual executed yaw turns. In addition to reporting the three angles separately, we also summed them (*β*; deg) to get an approximate sense of overall turning effort of the fin base. This method provides only an approximate measure because Euler angles depend on rotation order and are subject to magnitude distortions near the poles of Euler space (i.e. gimbal lock). However, given that all the fin motions were fairly small (<25°) and were zeroed near the equator, the sum of the three Euler angles likely provides a useful measure of overall turning effort of the fin base. All variables reported were taken from the frame of video where maximum total rotation was calculated. We compared the magnitude of inside fin rotation about three axes using a one-way ANOVA. Post-hoc Tukey's tests were used to compare variables if the ANOVA was significant. We report the mean±s.e. of the mean for all variables.

As a metric of turning performance, we calculated the angle of turning for each trial (*n*=9). A turn was defined as a change in heading in which one fin beat occurred. We measured the distance of the initial heading leading into the turn (*H_i_*), the distance of the final heading exiting the turn (*H_f_*) and calculated the hypotenuse (*H*) between the initial and final headings. Turning angle (*θ*; deg) was calculated using the law of cosines where
(1)
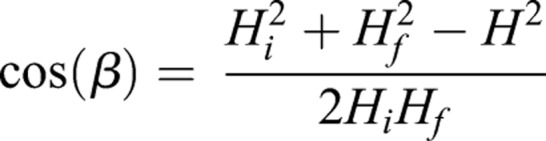
Porter et al. (2011). Angular velocity of the turn (*w_t_*; deg s^−1^) was calculated as the turning angle divided by the total time of the turn (*t*; s)
(2)
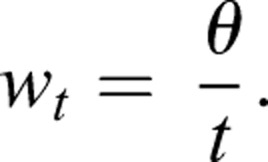
To estimate the hydrodynamics of the pectoral fin, we estimated drag and lift forces by digitizing the 2D fin shape and virtually rotating it. We created a 3D polygon object in Matlab using the *patch* function, which was then rotated using the 3D rotation values derived from the kinematics. The area of the fin to flow (*A*; m^2^) was calculated using the *polyarea* function by projecting the polygon object on to the XY plane, which was assumed to be orthogonal to flow. *A* was calculated for each frame based on the frame by frame rotation of the fin. Drag force (*F_d_*) was then estimated as
(3)
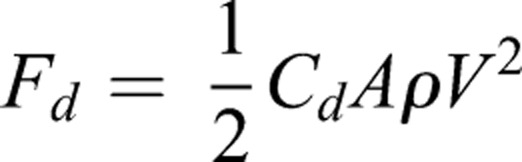
where *C_d_* is the drag coefficient of a streamlined shape based on wetted area,
(4)
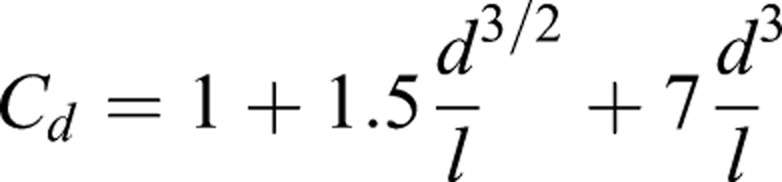
and *d* is the depth of the fin (m), *l* is the length (m), *ρ* is the density of saltwater at 10°C (kg m^−3^), and *V* is instantaneous linear velocity of the shark (m s^−1^) (Hoerner, 1965). These estimates treat the fin as a flat plate and do not consider conformational changes that are previously shown to affect force balance in shark dorsal fins (Maia and Wilga, 2013, 2016).

To describe the effect of fin rotation on turning performance, we used simple linear regressions to examine the relations among the turning angular velocity (*w_t_*) and pectoral fin rotation in all three axes (α), total pectoral fin rotation (*β*), the area of the fin to flow (*A*), and the estimated drag (*F_d_*).

## Supplementary Material

Supplementary information

## References

[BIO037291C1] Alexander R. M. (1965). The lift produced by the heterocercal tails of selachii. *J. Exp. Biol.* 43, 131-138.

[BIO037291C2] Blake R. W., Chatters L. M. and Domenici P. (1995). Turning radius of yellowfin tuna (Thunnus albacares) in unsteady swimming manoeuvres. *J. Fish. Biol.* 46, 536-538. 10.1111/j.1095-8649.1995.tb05994.x

[BIO037291C3] Brainerd E. L., Baier D. B., Gatesy S. M., Hedrick T. L., Metzger K. A., Gilbert S. L. and Crisco J. J. (2010). X-ray reconstruction of moving morphology (XROMM): Precision, accuracy, and application in comparative biomechanics research. *J. Exp. Zool. Part A Ecol. Genet. Physiol.* 313, 262-279. 10.1002/jez.589 20095029

[BIO037291C4] Brainerd E. L., Blob R. W., Hedrick T. L., Creamer A. T. and Müller U. K. (2017). Data management rubric for video data in organismal biology. *Integr. Comp. Biol.* 57, 33-47. 10.1093/icb/icx060 28881939 PMC5886321

[BIO037291C5] Camp A. L. and Brainerd E. L. (2014). Role of axial muscles in powering mouth expansion during suction feeding in largemouth bass (Micropterus salmoides). *J. Exp. Biol.* 217, 1333-1345. 10.1242/jeb.095810 24363416

[BIO037291C6] Camp A. L. and Brainerd E. L. (2015). Reevaluating musculoskeletal linkages in suction-feeding fishes with X-ray reconstruction of moving morphology (XROMM). *Integr. Comp. Biol.* 55, 36-47. 10.1093/icb/icv034 25972567

[BIO037291C7] Camp A. L., Scott B., Brainerd E. L. and Wilga C. D. (2017). Dual function of the pectoral girdle for feeding and locomotion in white-spotted bamboo sharks. *Proc. R. Soc. B.* 264, 20170847 10.1098/rspb.2017.0847 PMC5543220 28724735

[BIO037291C10] Daniel J. F. (1922). *The Elasmobranch Fishes*. University of California Press.

[BIO037291C13] Dial K. P., Kaplan S. R. and Goslow G. E. Jr. (1988). A functional analysis of the primary upstroke and downstroke muscles in the domestic pigeon (*Columba livia*) during flight. *J. Exp. Biol.* 134, 1-16. 3356961 10.1242/jeb.134.1.1

[BIO037291C14] Domenici P., Standen E. M. and Levine R. P. (2004). Escape manoeuvres in the spiny dogfish (*Squalus acanthias*). *J. Exp. Biol.* 207, 2339-2349. 10.1242/jeb.01015 15159438

[BIO037291C15] Drucker E. G. and Lauder G. V. (2001). Wake dynamics and locomotor function of the dorsal fin in teleost fishes: experimental analysis of wake forces in sunfish. *J. Exp. Biol.* 204, 2943-2958. 11551984 10.1242/jeb.204.17.2943

[BIO037291C16] Drucker E. G. and Lauder G. V. (2002). Wake dynamics and locomotor function in fishes: interpreting evolutionary patterns in pectoral fin design. *Integr. Comp. Biol.* 42, 997-1008. 10.1093/icb/42.5.997 21680381

[BIO037291C17] Drucker E. G. and Lauder G. V. (2003). Function of pectoral fins in rainbow trout: behavioral repertoire and hydrodynamic forces. *J. Exp. Biol.* 206, 813-826. 10.1242/jeb.00139 12547936

[BIO037291C65] Ferry L. and Lauder G. V. (1996). Heterocercal tail function in leopard sharks: a three-dimensional kinematic analysis of two models. *J. Exp. Biol.*, 199, 2253-2268. 9320170 10.1242/jeb.199.10.2253

[BIO037291C18] Fish F. E. (1997). Biological designs for enhanced maneuverability: analysis of marine mammal performance. Tenth International Symposium on Unmanned Untethered Submersible Technology: Proceedings of the Special Session on Bio-Engineering Research Related to Autonomous Underwater Vehicles 109-117.

[BIO037291C19] Fish F. E. (2002). Balancing requirements for stability and maneuverability in cetaceans. *Integr. Comp. Biol.* 42, 85-93. 10.1093/icb/42.1.85 21708697

[BIO037291C20] Fish F. E. and Lauder G. V. (2017). Control surfaces of aquatic vertebrates: active and passive design and function. *J. Exp. Biol.* 220, 4351-4363. 10.1242/jeb.149617 29187618

[BIO037291C21] Fish F. E. and Nicastro A. J. (2003). Aquatic turning performance by the whirligig beetle: constraints on maneuverability by a rigid biological system. *J. Exp. Biol.* 206, 1649-1656. 10.1242/jeb.00305 12682097

[BIO037291C22] Fish F. E. and Shannahan L. D. (2000). The role of the pectoral fins in body trim of sharks. *J. Fish Biol.* 56, 1062-1073. 10.1111/j.1095-8649.2000.tb02123.x

[BIO037291C64] Fish F. E., Kolpas A., Crossett A., Dudas M. A., Moored K. W. and Bart-Smith H. (2018). Kinematics of swimming of the manta ray: three-dimensional analysis of open water maneuverability. *J. Exp. Biol.* jeb-166041 29487154 10.1242/jeb.166041

[BIO037291C23] Flammang B. E. (2010). Function of the radialis muscle in shark tails. *J. Morphol.* 271, 340-352. 19827156 10.1002/jmor.10801

[BIO037291C24] Gilbert S. G. (1973). *Pictorial Anatomy of the Dogfish*. University of Washington Press.

[BIO037291C25] Goto T., Nishida K. and Nakaya K. (1999). Internal morphology and function of paired fins in the epaulette shark, *Hemiscyllium ocellatum*. *Ichthyol. Res.* 46, 281-287. 10.1007/BF02678514

[BIO037291C26] Harris J. E. (1936). The role of fins in the equilibrium of swimming fish. I. Wind-tunnel tests on a model of *Mustelus canis* (Mitchill)*. J. Exp. Biol.* 13, 474-493.

[BIO037291C28] Hedrick T. L. (2008). Software techniques for two- and three- dimensional kinematic measurements of biological and biomimetic systems. *Bioinspir. Biomim.* 3, 034001 10.1088/1748-3182/3/3/034001 18591738

[BIO037291C29] Hoerner S. F. (1965). Drag of streamlined shapes. In Fluid-dynamic drag: practical information on aerodynamic drag and hydrodynamic resistance. Brick Town, New Jersey.

[BIO037291C30] Hoffmann S. L., Warren S. M. and Porter M. E. (2017). Regional variation in undulatory kinematics of two hammerhead species, the bonnethead (*Sphyrna tiburo*) and the scalloped hammerhead (*Sphyrna lewini*). *J. Exp. Biol.* 220, 3336-3343. 10.1242/jeb.157941 28705829

[BIO037291C31] Jackson B. E., Dennis E. J., Ray D. D. and Hedrick T. L. (2016). 3D for the people: mutli-camera motion capture in the field with consumer grade cameras and open source software. *Biol. Open* 2016, 1334-1342. 10.1242/bio.018713 PMC5051647 27444791

[BIO037291C32] Jimenez Y. E., Camp A. L., Grindall J. D. and Brainerd E. L. (2018). Axial morphology and 3D neurocranial kinematics in suction-feeding fishes. *Biol. Open* 7, p.bio036335 10.1242/bio.036335 PMC6176947 30237249

[BIO037291C33] Kajiura S. M., Forni J. B. and Summers A. P. (2003). Maneuvering in juvenile carcharhinid and sphyrnid sharks: the role of the hammerhead shark cephalofoil. *Zoology* 106, 16-28. 10.1078/0944-2006-00086 16351888

[BIO037291C34] Kato N., Liu H., Morikawa H. (2004). Biology inspired precision maneuvering of underwater vehicles. In *Bio-Mechanisms of Swimming and Flying* (ed. Kato N., Ayers J. and Morikawa H.), pp. 111-125. Springer Science & Business Media. Tokyo: Springer.

[BIO037291C35] Knörlein B. J., Baier D. B., Gatesy S. M., Laurence-Chasen J. D. and Brainerd E. L. (2016). Validation of XMALab software for marker-based XROMM. *J. Exp. Biol.* 219, 3701-3711. 10.1242/jeb.145383 27655556

[BIO037291C36] Lauder G. V. (2015). Flexible fins and fin rays as key transformations in ray-finned fishes. In *Great Transformations in Vertebrate Evolution* (ed. Dial K. P., Shubin N. and Brainerd E. L.), pp. 31-45. Chicago: University of Chicago Press.

[BIO037291C37] Lauder G. V. and Drucker E. G. (2004). Morphology and experimental hydrodynamics of fish fin control surfaces. *IEEE J. Ocean. Eng.* 29, 556-571. 10.1109/JOE.2004.833219

[BIO037291C66] Leardini A., Chiari L., Della Croce U. and Cappozzo A. (2005). Human movement analysis using stereophotogrammetry: Part 3. Soft tissue artifact assessment and compensation. *Gait & posture* 21, 212-225. 15639400 10.1016/j.gaitpost.2004.05.002

[BIO037291C68] Liem K. F and Summers A. P (1999). Muscular system. In *Sharks, skates, and rays: The biology of elasmobranch fishes* (ed. Hamlett, William C.), pp. 93-114 Johns Hopkins University Press, Baltimore.

[BIO037291C67] Lowe C. (1996). Kinematics and critical swimming speed of juvenile scalloped hammerhead sharks. *J. Exp. Biol.* 199, 2605-2610. 10.1242/jeb.149617 9320537

[BIO037291C38] Maia A. M. R. and Wilga C. A. D. (2013). Function of dorsal fins in bamboo shark during steady swimming. *Zoology* 116, 224-231. 10.1016/j.zool.2013.05.001 23830781

[BIO037291C39] Maia A. M. R. and Wilga C. A. D. (2016). Dorsal fin function in spiny dogfish during steady swimming. *J. Zool.* 298, 139-149. 10.1111/jzo.12300

[BIO037291C40] Maia A. M. R., Wilga C. A. D. and Lauder G. V. (2012). Biomechanics of locomotion in sharks, rays, and chimeras. In *Biology of Sharks and their Relatives* (ed. Carrier J. C., Musik J. A. and Heithaus M. R.), 2nd edn, pp. 125-151. Boca Raton: CRC Press.

[BIO037291C41] Marinelli W. and Strenger A. (1959). Superklasse: Gnathostomata. (Kiefermäuler). Klasse: Chondrichthyes. (Knorpelfische). In *Vergleichende Anatomie und Morphologie der Wirbeltiere. II*.

[BIO037291C42] Moss S. A. (1972). Nurse shark pectoral fins: an unusual use. *Am. Mid. Nat.* 88, 496-497. 10.2307/2424384

[BIO037291C44] Nursall J. R. (1962). Swimming and the origin of paired appendages. *Am. Zool.* 2, 127-141. 10.1093/icb/2.2.127

[BIO037291C45] Oliver S. P., Turner J. R., Gann K., Silvosa M. and Jackson T. D. U. (2013). Thresher sharks use tail-slaps as a hunting strategy. *PLoS ONE* 8, e67380 10.1371/journal.pone.0067380 23874415 PMC3707734

[BIO037291C46] Porter M. E., Roque C. M. and Long J. H. (2009). Turning maneuvers in sharks: predicting body curvature from vertebral morphology. *J. Morph.* 270, 954-965. 10.1002/jmor.10732 19248153

[BIO037291C47] Porter M. E., Roque C. M. and Long J. H. (2011). Swimming fundamentals: turning performance of leopard sharks (Triakis semifasciata) is predicted by body shape and postural reconfiguration. *Zoology* 114, 348-359. 10.1016/j.zool.2011.06.001 21982409

[BIO037291C48] Pridmore P. A. (1994). Submerged walking in the epaulette shark *Hemiscyllium ocellatum* (Hemiscyllidae) and its implications for locomotion in rhipidistian fish and early tetrapods. *ZACS Zool.* 98, 278-297.

[BIO037291C49] Rohr J. J. and Fish F. E. (2002). Strouhal numbers and optimization of swimming by odontocete cetaceans. *J. Exp. Biol.* 207, 1633-11642. 10.1242/jeb.00948 15073196

[BIO037291C50] Ros I. G., Bassman L. C., Badger M. A., Pierson A. N. and Biewener A. A. (2011). Pigeons steer like helicopters and generate down-and upstroke lift during low speed turns. *Proc. Nat. Acad. Sci. USA* 108, 19990-19995. 10.1073/pnas.1107519108 22123982 PMC3250151

[BIO037291C51] Seamone S., Blaine T. and Higham T. E. (2014). Sharks modulate their escape behavior in response to predator size, speed, and approach orientation. *Zoology* 117, 377-382. 10.1016/j.zool.2014.06.002 25041843

[BIO037291C52] Sellers W. I. and Hirasaki E. (2014). Markerless 3D motion capture for animal locomotion studies. *Biol. Open* 3, 656-668. 10.1242/bio.20148086 24972869 PMC4154302

[BIO037291C53] Tangorra J. L., Lauder G. V., Hunter I. W., Mittal R., Madden P. G. A. and Bozkurttas M. (2010). The effect of fin ray flexural rigidity on the propulsive forces generated by a biorobotic fish pectoral fin. *J. Exp. Biol.* 213, 4043-4054. 10.1242/jeb.048017 21075946

[BIO037291C54] Tobalske B. W. (2010). Hovering and intermittent flight in birds. *Bioinsp. Biomim.* 5, 045004 10.1088/1748-3182/5/4/045004 21098953

[BIO037291C55] Tobalske B. W., Hedrick T. L. and Biewener A. A. (2003). Wing kinematics of avian flight across speeds. *J. Avian Biol.* 34, 177-184. 10.1034/j.1600-048X.2003.03006.x

[BIO037291C56] Tomita T., Tanaka S., Sato K. and Nakaya K. (2014). Pectoral fin of the megamouth shark: skeletal and muscular systems, skin histology, and functional morphology. *PLoS ONE* 9, e86205. 24465959 10.1371/journal.pone.0086205 PMC3897653

[BIO037291C58] Webb P. W. (1984). Body form, locomotion, and foraging in aquatic vertebrates. *Amer. Zool.* 24, 107-120. 10.1093/icb/24.1.107

[BIO037291C60] Webb P. W. and Weihs D. (2015). Stability versus maneuvering: challenges for stability during swimming by fishes. *Int. Comp. Biol.* 55, 753-764. 10.1093/icb/icv053 26002562

[BIO037291C61] Wilga C. D. and Lauder G. V. (1999). Locomotion in sturgeon: function of the pectoral fins. *J. Exp. Biol.* 202, 2413-2432. 10460730 10.1242/jeb.202.18.2413

[BIO037291C62] Wilga C. D. and Lauder G. V. (2000). Three-dimensional kinematics and wake structure of the pectoral fins during locomotion in leopard sharks *Triakis semifasciata*. *J. Exp. Biol.* 203, 2261-2278. 10887066 10.1242/jeb.203.15.2261

[BIO037291C63] Wilga C. D. and Lauder G. V. (2001). Functional morphology of the pectoral fins in bamboo sharks, *Chiloscyllium plagiosum*: Benthic vs. pelagic station holding. *J. Morph.* 249, 195-209. 10.1002/jmor.1049 11517464

