# Supplementary figures and images for: Three-dimensional movements of the pectoral fin during yaw turns in the Pacific spiny dogfish, Squalus suckleyi
Source: Biol Open. 2018 Dec 24;8(1):bio037291. doi: 10.1242/bio.037291 (PMC6361209; doi:10.1242/bio.037291)

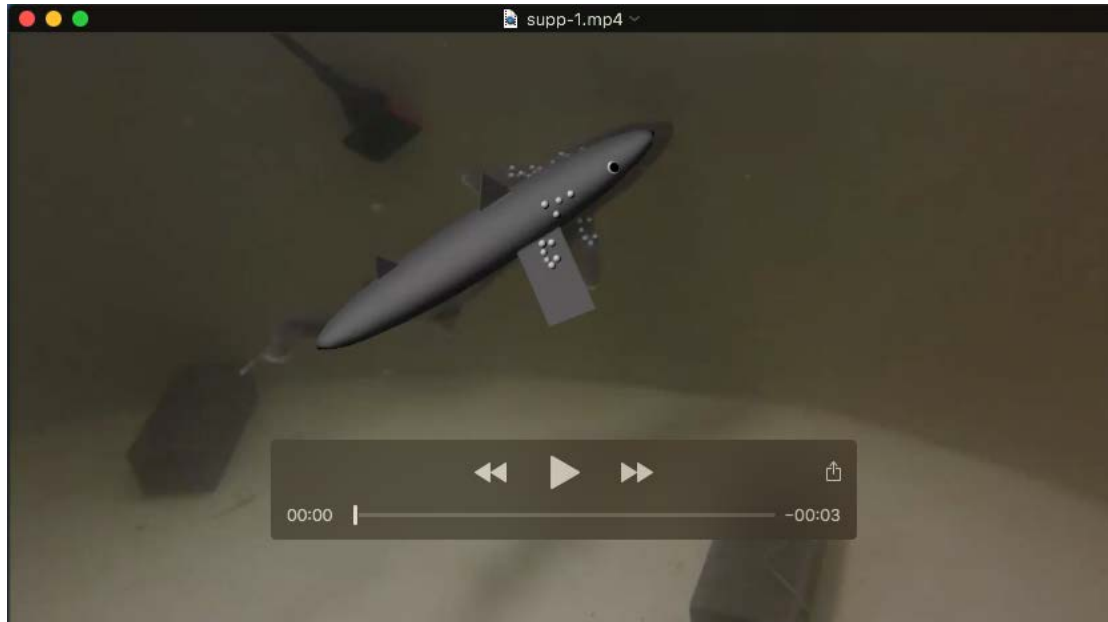

Movie 1: 3D reconstruction of body and fin movement during yaw turning.

Supplement: Supplementary information [file biolopen-8-037291-s1.pdf]
